# Rewiring Receptor Activation: Mechanistic Insights into Toggle Switch Modulation by 25CN-NBx Compounds

**DOI:** 10.1021/acschemneuro.6c00023

**Published:** 2026-02-18

**Authors:** Vito F. Palmisano, Micaela Vidal−Sánchez, Juan J. Nogueira

**Affiliations:** † Department of Chemistry, 16722 Universidad Autonoma de Madrid, 28049 Madrid, Spain; ‡ International Foundation Big Data and Artificial Intelligence for Human Development, 40121 Bologna, Italy; § Department of Chemistry, Universidad Autonoma de Madrid, 28049 Madrid, Spain; ∥ IADCHEM, Institute for Advanced Research in Chemistry, Universidad Autonoma de Madrid, 28049 Madrid, Spain

**Keywords:** 5-hydroxytryptamine 2A receptor (5-HT_2A_R), 25CN-NBx compounds, *N*-benzyl, G protein-coupled receptor (GPCR), van der Waals (vdw)

## Abstract

The development of new treatments for neuropsychiatric disorders relies on a deeper understanding of the molecular mechanisms behind psychedelic compounds, particularly how they differentiate between hallucinogenic and antidepressant effects. In this study, we explore by molecular dynamics simulations a series of 25CN-NBx compounds bound to the 5-HT_2A_ receptor. The simulations uncover that bulky substitutions on the *N*-benzyl ring cause a significant shift in the position of W336, a key toggle switch residue known to influence receptor activation and thought to play a crucial role in mediating psychedelic signaling. This result is confirmed through potential of mean force calculations along the toggle switch’s dihedral angle. End-state free energy calculations align closely with experimental data, showing that 25CN-NB-2-OH-3-Me and 25CN-NB-2-OH-5-MeO have the highest and lowest affinities, respectively, for the 5-HT_2A_ receptor. Further analysis indicates that when W336 adopts its negative dihedral state, it establishes stronger van der Waals interactions, stabilizing its contacts with residues F332 and I163—key players previously linked to receptor activation. Our findings provide a framework for understanding receptor activation and can be extended to other G protein-coupled receptors where the toggle switch is central to signal activation.

## Graphical abstract

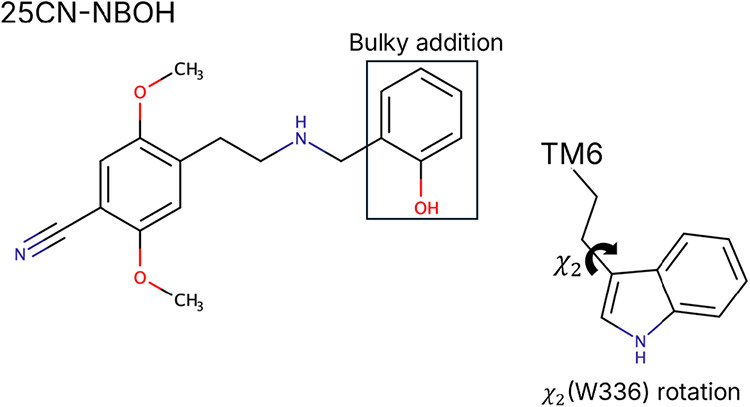

## Introduction

One of the central questions in the neuropsychopharmacology of psychedelic compounds is whether the acute hallucinatory effects can be separated from the rapid and long-lasting antidepressant effects at the downstream signaling level.^1^ This question drives current research efforts toward understanding the role of the 5-hydroxytryptamine 2A receptor (5-HT_2A_R), which is known to mediate both hallucinatory and antidepressant effects,^2–5^ although recent findings also suggest that neural plasticity could be promoted independently of the 5-HT_2A_R.^6^ Serotonergic psychedelics, derived from various chemotypes (e.g., tryptamines, phenethylamines, and ergotamines), activate the 5-HT_2A_R with different potency and efficacy.^7^ Indeed, 5-HT_2A_R agonists typically activate both the G_q/11_ pathway^8,9^ and β-arrestin2 translocation,^10,11^ with most of them exhibiting similar activation profiles to the balanced endogenous agonist 5-HT or serotonin.^12^ Recent advances in the structure-based discovery of 5-HT_2A_R have led to the development of compounds that act as biased agonists, capable of stabilizing the receptor in conformations that preferentially favor one downstream signaling pathway over the other.^13^ Kaplan et al.,^14^ through virtual screening of several million molecules, identified two compounds that produced antidepressant-like effects in mice without hallucinogenic properties, showing a bias toward G_q/11_ activation over β-arrestin2 recruitment. In contrast, Wang et al.^15^ identified two other compounds that retained antidepressant-like effects in mice without inducing a hallucinogenic response and showed a bias toward β-arrestin2 signaling. In this latter case, a high transduction efficiency of 5-HT_2A_R agonists in both G_q/11_ and β-arrestin2 recruitment was required to induce hallucinogenic effects, while a low transduction efficiency of 5-HT_2A_R β-arrestin2 bias was sufficient to achieve antidepressant-like effects.

While developing new chemotypes targeting the 5-HT_2A_R, recent years have seen significant advancements in the structure–activity relationships of compounds derived from mescaline (Figure 1A) and 2,5-Dimethoxy-4-iodoamphetamine (DOI) (Figure 1B).^7,16^ Notably, the incorporation of an *N*-benzyl group at the ethylamine domain has led to the synthesis of a class of compounds with remarkable affinity and potency for the 5-HT_2A_R.^17,18^

**Figure 1:**
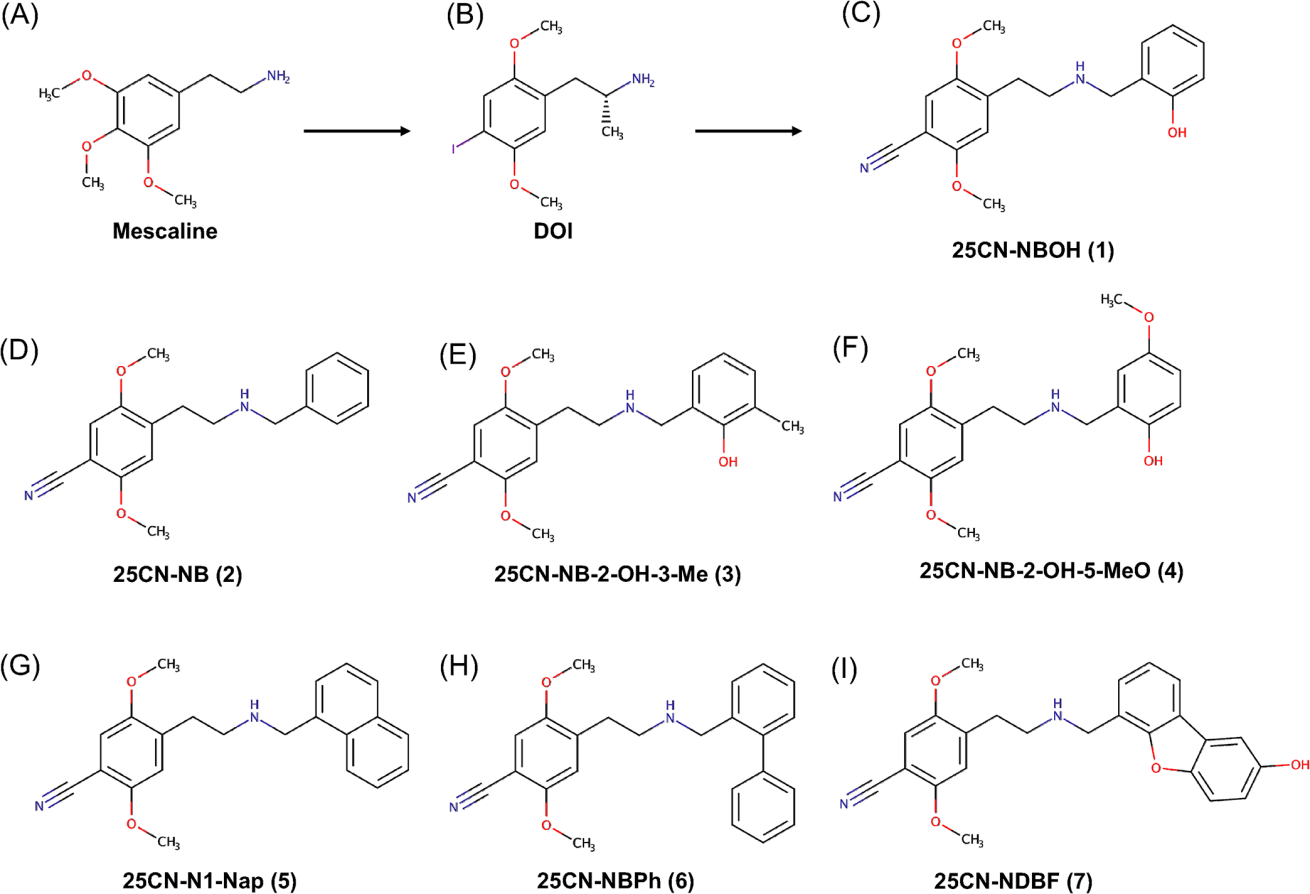
Chemical structure of (A) mescaline, (B) DOI, (C) 25CN-NBOH, (D) 25CN-NB, (E)­25CN-NB-2-OH-3-Me, (F) 25CN-NB-2-OH-5-MeO, (G) 25CN-N1-Nap, (H) 25CN-NBPh, (I) 25CN-NDBF.

Among these DOI derivatives, 25CN-NBOH **(1)** (Figure 1C) exhibits high binding affinity for the 5-HT_2A_R, with significantly lower affinity for the other two subtypes, 5-HT_2B_R and 5-HT_2C_R.^19–21^ Recently, **(1)** was crystallized bound to the 5-HT_2A_R, revealing a new binding pose compared to the crystallized structure of the 5-HT_2A_R bound to LSD. In this pose, the 2-OH group is inserted deeply into the orthosteric binding pocket, interacting with a novel region of the receptor between helices TM3 and TM6 and forming key interactions with the indole ring of W336 (Figure 2A).^10^ This residue, known as the ”toggle switch”, has been proposed to control G protein-coupled receptor (GPCR) activation during signal transduction,^22^ and a comparison with other 5-HTRs bound to other chemotypes reveals that the crystallized structure of the 5-HT_2A_R bound to **(1)** displays the largest change in displacement of W336.^23,24^

**Figure 2:**
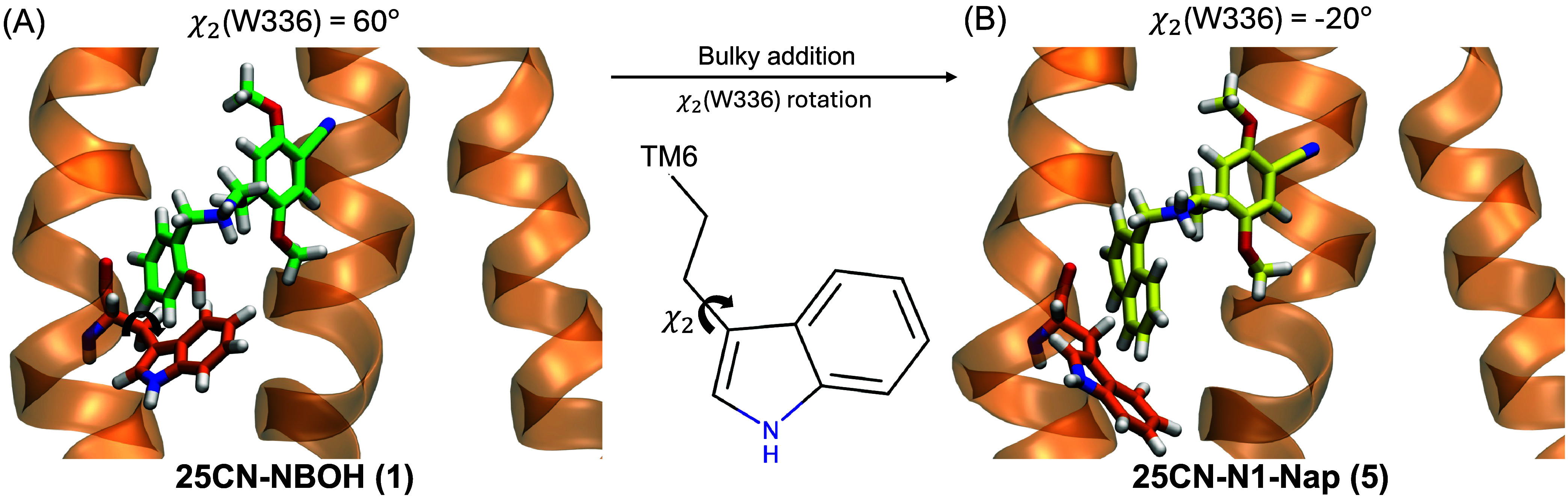
Representation of a portion of the 5-HT_2A_ receptor (orange) highlighting W336 to illustrate the transition from (A) a positive value of the χ_2_(W336) dihedral angle when interacting with 25CN-NBOH **(1)**, where the indole core of the residue points toward the extracellular medium, to (B) a negative value of the χ_2_(W336) dihedral angle when interacting with 25CN-N1-Nap **(5)**, where the indole core of the residue points toward the intracellular medium.

In 2023, Wallach et al.^12^ conducted a study on various 25N-NBx compounds, demonstrating that the addition of bulky groups results in a substantial reduction in 5-HT_2A_R G_q/11_ efficacy while preserving 5-HT_2A_R β-arrestin2 efficacy. In addition, it was shown that changes in the electron density at the *N*-benzyl ring highly influence the 5-HT_2A_R affinity (Figure 1D–F). Induced fit molecular docking and molecular dynamics (MD) simulations of these bulky compounds, 25CN-N1-Nap **(5)** (Figure 1G) and 25CN-NBPh **(6)** (Figure 1H), show a substantial change in the displacement of the χ_2_ dihedral angle of W336 (χ_2_(W336)) from positive to negative values, driving the indole core of the residue toward the inner side of the 5-HT_2A_R (Figure 2). These β-arrestin2 biased compounds demonstrated a lack of hallucinogenic potential, and a comprehensive assay of 25N-NBx derivatives revealed that when G_q/11_ activation exceeds 70% efficacy, hallucinogenic effects are observed in mice. In contrast, β-arrestin2 activation alone, as seen with 25N-NBx compounds containing bulky substituents, does not appear to be sufficient to induce such effects. A correlation emerges between the weak G_q/11_ activation and the negative values of χ_2_(W336), highlighting the importance of developing a computational protocol capable of predicting the most stable orientation of toggle switch upon *N*-benzyl additions to the 25CN-NBx compounds.

To this end, in this computational study, we applied MD simulations in combination with umbrella sampling (US) and molecular mechanics generalized Born surface area (MMGBSA) free energy calculations to obtain the potential of mean force (PMF) along the χ_2_(W336) upon different *N*-benzyl additions for a set of 25CN-NBx compounds (Figure 1C–I). Given that during unbiased MD simulations of some compounds, W336 transitioned from its initial state with χ_2_(W336) = 60°, as in the 5-HT_2A_R-**(1)** complex, to a state where the indole core pointed deep into the receptor, corresponding to negative values of χ_2_(W336), we used US to obtain the PMF along the χ_2_(W336) and identify the local minima for each compound. The identified minima were correlated with the G_q_ efficacy data reported by Wallach et al., demonstrating that the addition of bulky substituents to the *N*-benzyl group stabilizes the toggle switch in conformations with negative χ_2_(W336) values. In this orientation, where the indole core of W336 points toward the intracellular side of the receptor, the residue establishes strong van der Waals interactions with I163 and F332-two residues previously implicated in a conserved motif that undergoes conformational changes during receptor activation.^25^ These findings offer insight into how allosteric changes are propagated within the active state of the 5-HT_2A_R, and highlight a transferable strategy for studying other GPCRs in which the TM6 toggle switch plays a central role in receptor activation.

## Results and Discussion

### Molecular Dynamics Simulations: Spontaneous Toggle Switch Rotation

To validate the stability of **(1)-(7)** inside the orthosteric binding pocket, four independent replicas of 1 μs production run were conducted for each compound. The RMSD of the ligands with respect to the aligned 5-HT_2A_R shows that all the compounds remained tightly bound in the orthosteric binding pocket maintaining the initial pose throughout the full 4 μs simulation with an average value of approximately 1.5 Å (Figure 3).

**Figure 3:**
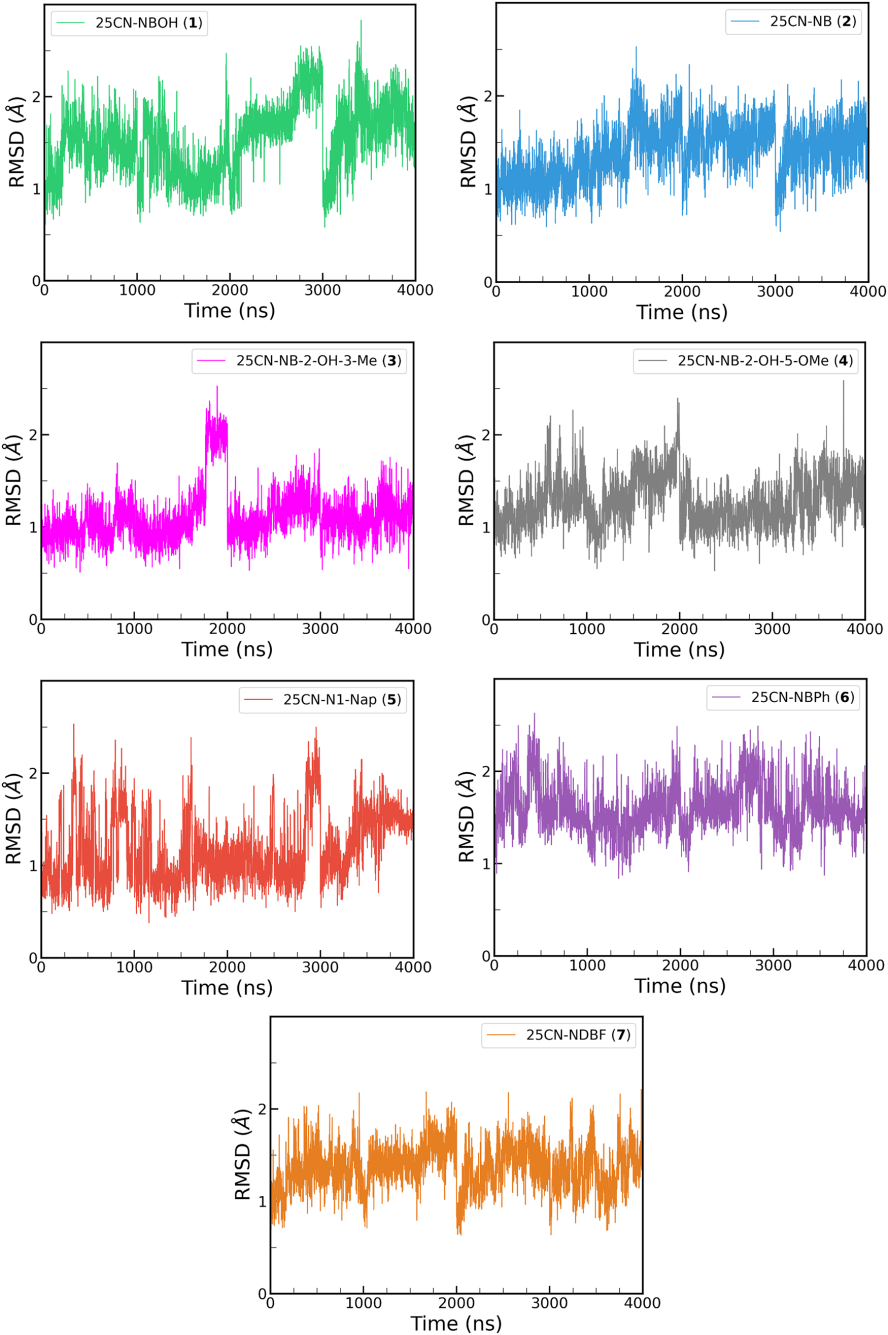
RMSD of **(1)-(7)** with respect to the non hydrogen atoms of the aligned 5-HT_2A_ receptor for the 4 μs of MD simulations.

Interestingly, during the dynamics of **(3)**, **(4)**, **(5)**, **(6)**, and 25CN-NDBF **(7)**, the χ_2_(W336) dihedral angle naturally jumped from its initial value of approximately 60–80° to negative values of approximately −20° (Figure 4). Given that in the apo-structure χ_2_(W336) exhibits a value of approximately 100°, with its indole core oriented toward the extracellular domain,^25^ and shifts to around 60° in the crystal structure bound to **(1)**, we chose to use χ_2_(W336) as a reaction coordinate for US simulations to obtain a converged PMF and identify the most stable conformation for each of the psychedelic compounds. The goal is to find a relationship between the ligand structure and the binding pose, especially regarding the W336 oritentation and its potential role in the activation of a particular biological route.

**Figure 4:**
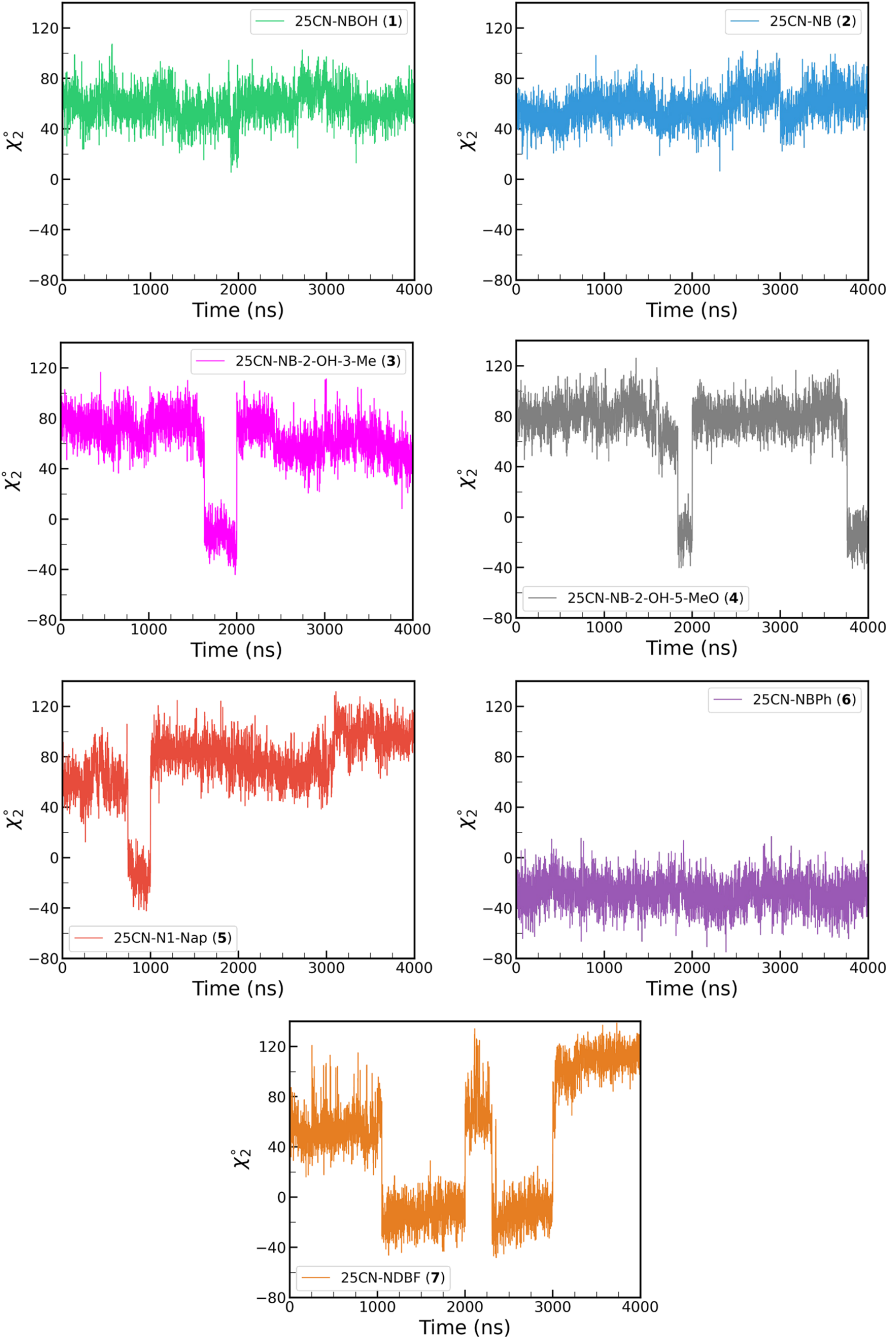
Distribution of the χ_2_(W336) dihedral angle along the 4 μs of cMD simulation for **(1)-(7)**.

### Identification of Two Toggle Switch Conformations by Umbrella Sampling Simulations

The US simulations validated that the addition of *N*-benzyl substituents, particularly bulky substituents as in **(5)**,**(6)**, and **(7)**, forces the toggle switch W336 to orient its indole core toward the inner part of the receptor, with negative χ_2_(W336) values (Figure 5).

**Figure 5:**
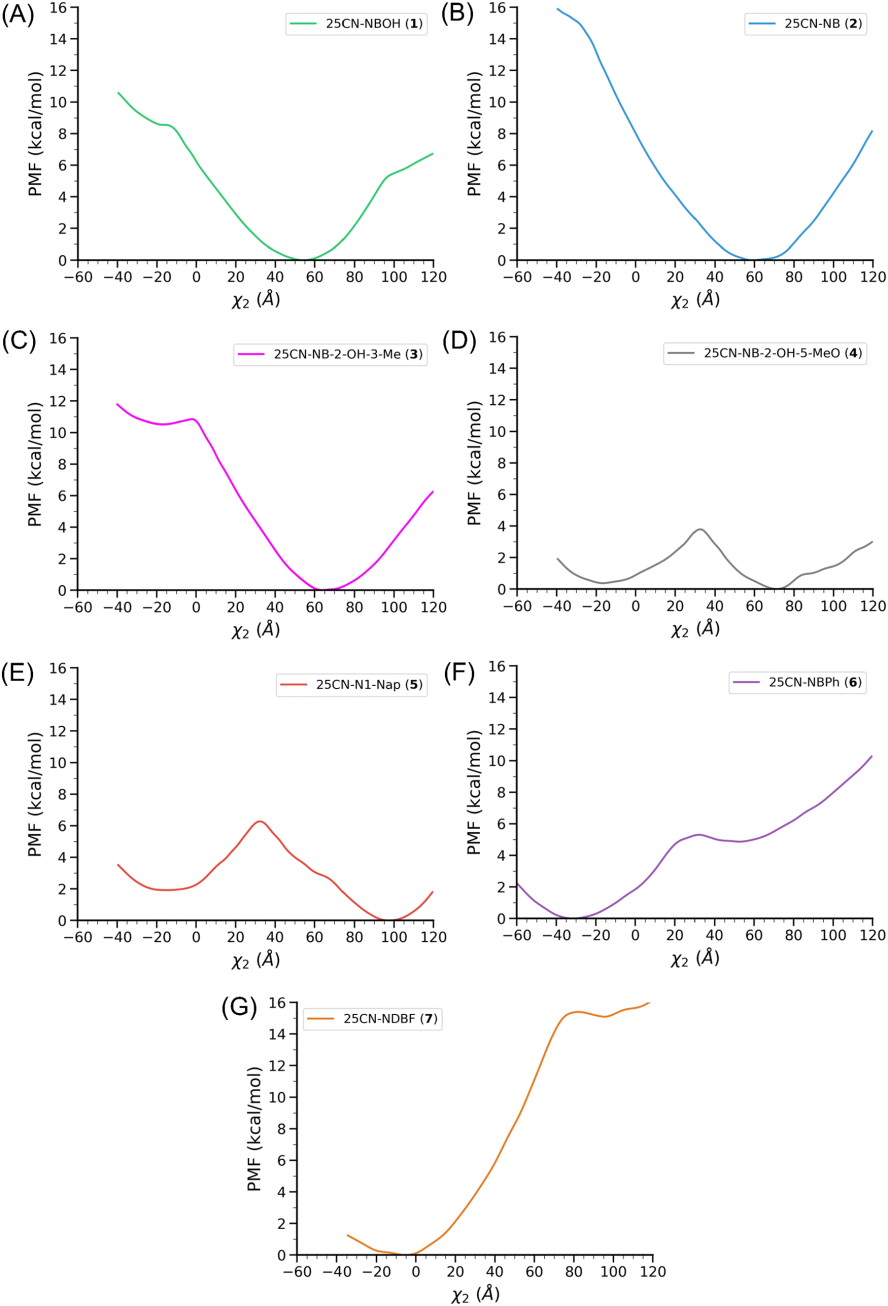
Potential of mean force (PMF) along the χ_2_(W336) dihedral angle for **(1)-(7)**.

Starting from **(1)** (Figure 5A), we begin our comparison with **(2)**, **(3)**, and **(4)** to observe the effect of −2-OH, −2-OH-3-Me, and −2-OH-5-MeO additions to the ring (Figure 5B-D). In **(1)** and **(2)**, only one evident minimum is present at χ_2_(W336) = 60°, with a small change in the PMF upon −2-OH addition at χ_2_(W336) = −20°. Instead, the addition of −2-OH-3-Me in **(3)** induces a pronounced formation of a second minimum at χ_2_(W336) = −20°, 11 kcal/mol higher in energy than the minimum displayed at χ_2_(W336) = 60°. Finally, the addition of −2-OH-5-MeO in compound **(4)** induces a substantial change in the PMF, pushing the minimum at χ_2_(W336) = −20° at the same energy as χ_2_(W336) = 60°. Indeed, both −2-OH-3-Me and −2-OH-5-MeO additions show experimentally a reduced G_q/11_ efficacy, indicating that this reduction in activity can be well correlated with the negative χ_2_(W336) values and the indole core of the toggle switch projected toward the inner side of the receptor.^12^

As highlighted earlier, Wallach et al., motivated by the observed reduction in G_q/11_ efficacy upon C_3′_ addition, further explored various substituents on the *N*-benzyl ring, increasing the bulk on the side of the molecule that extends toward an unexplored region of the orthosteric binding pocket. The PMFs from the US simulations for **(5)**, **(6)**, and a newly synthesized compound, **(7)**, all of which exhibited low G_q/11_ efficacy,^12,26^ show minima at negative χ_2_(W336) values. The **(5)** species (Figure 5E) exhibits two clear minima, with the deepest one at χ_2_(W336) = 100°, and the other at χ_2_(W336) = −15°, with a minor energy difference of 2 kcal/mol. Conversely, **(6)** (Figure 5F) displays the deepest minimum at χ_2_(W336) = −30° and the other at χ_2_(W336) = 60°, which is 6 kcal/mol higher in energy. Finally, 25CN-NDBF **(7)** (Figure 5G) presents a clearly different scenario with one minimum at χ_2_(W336) = 100°, similar to 25CN-N1-Nap **(5)**, but located 16 kcal/mol higher in energy than the global minimum at χ_2_(W336) = −5°. The binding of **(7)** also demonstrated that a dibenzofuran addition is well tolerated in the inner side of the 5-HT_2A_R, indicating that large bulky substituents are well accommodated in that region of the 5-HT_2A_R. The US simulations provide a reliable tool for precisely estimating the conformational changes of the toggle switch W336 and can be extended to study other GPCR receptors.

### Binding Free Energy Calculations

#### *N*-Benzyl Polar Substituents Addition

The crystallized structure of 5-HT_2A_R bound to **(1)** reveals a hydrogen bond between the 2-OH substituent and polar residues in TM3 of the receptor,^10^ which is suggested to contribute to its increased affinity^10,27^ and is further analyzed in the pairwise ligand-residue decomposition section. Based on this observation, we compared the binding affinity of the unsubstituted *N*-benzyl compound **(2)** with the −2-OH derivative **(1)**, as well as with two additional compounds that were experimentally reported to have the highest and lowest affinity for the 5-HT_2A_R, namely **(3)** and **(4)**.^12^ Our results confirm that compound **(3)** exhibits the most favorable Δ*G*_tot_, followed by **(1)** and **(2)**, whereas **(4)** shows the least favorable Δ*G*_tot_, with a difference of 9.6 kcal/mol relative to the highest-affinity compound. The primary factor that differentiates the binding of **(3)** is its more favorable Δ*G*_vdw_, suggesting that the −3-Me substitution enhances hydrophobic contacts and stabilizes specific interactions within the orthosteric binding pocket. In contrast, the other three compounds display very similar Δ*G*_vdw_ values, indicating that the −2-OH and −5-OMe groups do not substantially influence the hydrophobic environment of the pocket, see Table 1.

**Table 1:** Binding Free Energy (Δ*G*_tot_) of **(1)-(4)**/ 5-HT_2A_R and Decomposition into Ligand/Receptor van der Waals (Δ*G*_vdw_) and Electrostatic (Δ*G*_el_) Interactions, and Complex/Solvent Polar (Δ*G*_polar_) and Non Polar (Δ*G*_np_) Interactions in kcal/mol

compounds	Δ*G*_vdw_	Δ*G*_el_	Δ*G*_pol_	Δ*G*_el+pol_	Δ*G*_np_	Δ*G*_tot_
25CN-NBOH **(1)**	–53.7	–106.3	98.2	–8.1	–6.8	–68.6
25CN-NB **(2)**	–54.9	–116.7	111.3	–5.4	–6.7	–67.0
25CN-NB-2-OH-3-Me **(3)**	–60.7	–109.4	106.4	–3.0	–7.1	–70.9
25CN-NB-2-OH-5-MeO **(4)**	–55.8	–107.9	109.5	+1.6	–7.0	–61.3

Notably, all three substitutions in **(1)**, **(3)**, and **(4)** lead to a reduction in the raw Coulombic term (Δ*G*_el_) compared to the unsubstituted compound **(2)**, which is about 10 kcal/mol more favorable. At first sight, this would suggest that **(2)** engages in the strongest electrostatic interactions with the receptor. However, this apparent advantage is largely offset by its high polar solvation penalty (Δ*G*_pol_ = 111.3 kcal/mol). In other words, while **(2)** forms strong protein–ligand electrostatics, it also pays the greatest cost for losing water–ligand interactions upon binding. By contrast, compound **(1)** shows a somewhat weaker Δ*G*_el_, but this is compensated by the lowest desolvation penalty (98.2 kcal/mol), resulting in the most favorable net electrostatic energy. Compounds **(3)** and **(4)** are intermediate situations: **(3)** incurs a moderate desolvation penalty (106.4 kcal/mol), while **(4)** experiences a net electrostatic contribution that is even slightly unfavorable (1.6 kcal/mol), since the expected hydrogen-bonding benefits of its −2-OH and −5-OMe groups with the protein do not outweigh the cost of desolvation. Altogether, these results indicate that the balance between Coulombic interactions with the protein and desolvation energy, rather than either term alone, determines the effective electrostatic contribution to binding. The nonpolar contribution to the solvation energy, Δ*G*_np_, remains similar across all compounds and appears to play a less discriminating role in the observed differences.

Each binding energy term was further decomposed into pairwise ligand–residue contributions (Figure 6). Consistent with the crystal structure reported by Kim et al.,^10^ all the major interactions are retained in **(1)**. In TM3, D155 provides the dominant contribution through a salt bridge with the ligand’s tertiary amine, while V156 and S159 contribute additional stabilization via alkyl−π and hydrogen-bonding interactions, respectively. Further stabilization arises from the hydrophobic cage in TM6 and TM7, formed by W336, F339, F340, G369, and T370. Interestingly, **(1)**, **(3)**, and **(4)** display slightly weaker Δ*G*_el_ values at S159 compared to **(2)**, suggesting that hydrogen bonding at this site may partially compete with the salt bridge to D155, thereby reducing the net electrostatic contribution. This observation complements our earlier analysis showing that compounds **(1)**, **(3)**, and **(4)** pay a lower desolvation penalty but do not necessarily gain stronger direct Coulombic interactions. The substitution at the C_3′_ position in **(3)** further explains its superior binding. The −3-Me group enhances van der Waals stabilization, with contributions distributed across all major residues in the hydrophobic cage. Along with compound **(4)**, **(3)** also shows strengthened interactions with the toggle switch residue W336, consistent with the conformational adaptation observed in our MD simulations. These results confirm that the C_3′_ addition increases affinity for the 5-HT_2A_R primarily through enhanced hydrophobic packing of the *N*-benzyl ring within the aromatic cage, in agreement with the trends in Δ*G*_vdw_ described above.

**Figure 6:**
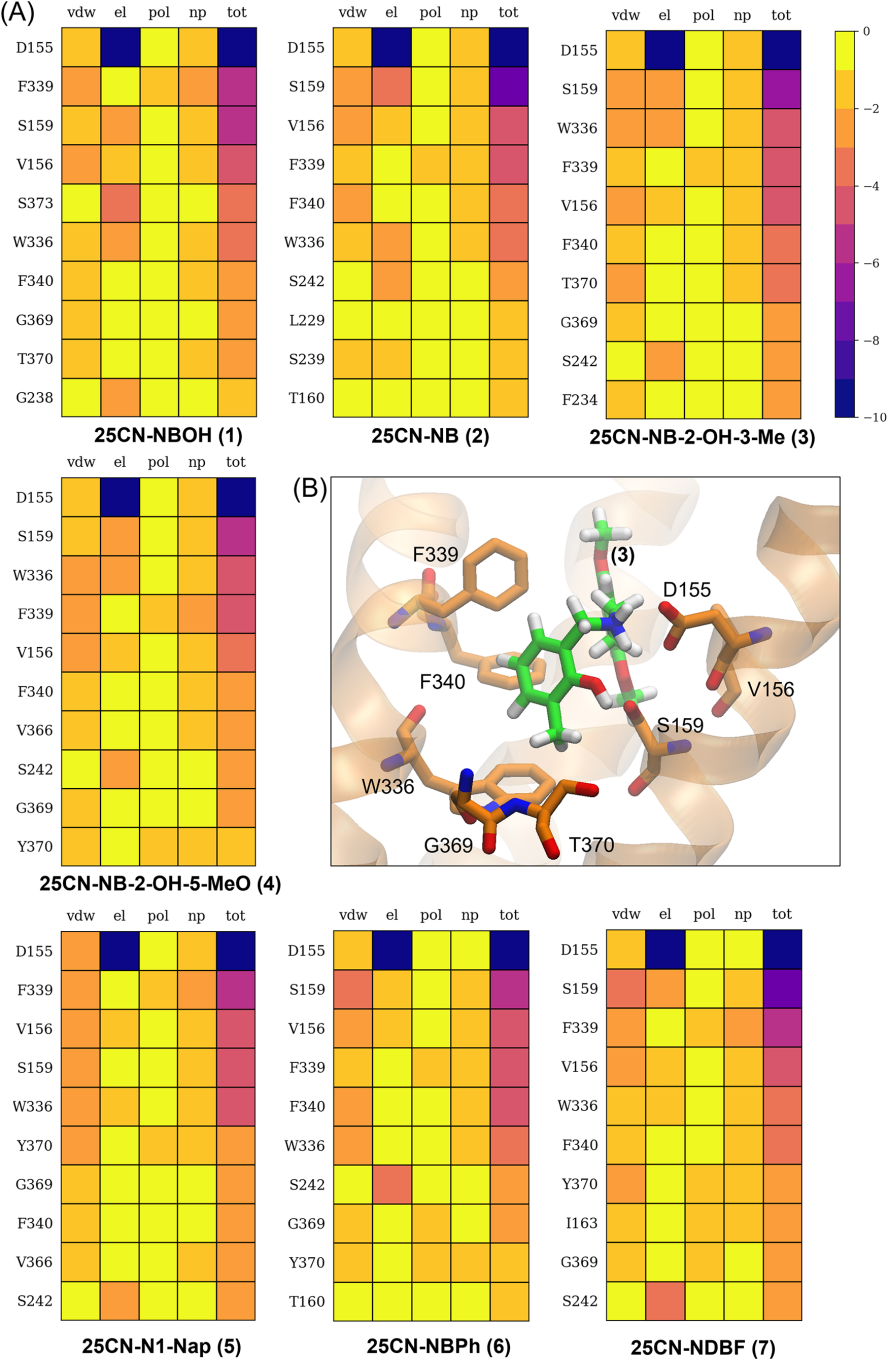
(A) Pairwise residue decomposition of the binding free energy for **(1)-(7)**. Each residue contribution is in turn decomposed into ligand/protein van der Waals (vdw) and electrostatic (el) contributions and polar (pol) and nonpolar (np) solvation contributions to the total residue binding energy (tot). (B) Binding pose of **3** in the OBP of 5-HT2AR with the eight residues that contribute most to the binding energy and the corresponding helices (S3, S6, S7).

#### *N*-Benzyl Bulky Substituents Addition

The addition of bulky substituents, as in **(5)**, **(6)**, and **(7)**, induces an increase in Δ*G*_vdw_, with **(7)** displaying the highest increase, which is mirrored in the highest Δ*G*_tot_ (Table 2). The electrostatic interactions are more favorable for **(6)**, but it also incurs the highest Δ*G*_pol_ penalty, which is 6 kcal/mol higher compared to the other two compounds, resulting in the least favorable Δ*G*_pol_. The pairwise ligand-residue free energy decomposition reveals relatively similar interactions among the three compounds. However, for **(7)**, the Δ*G*_vdw_ values are more favorable and evenly distributed among the residues in the pocket, attributed to the dibenzofuran substituent, which penetrates deeper into the inner part of the 5-HT_2A_R. In addition to interactions within the hydrophobic cage of TM6, primarily involving W336, F339, and F340, these interactions extend to residues in TM7, particularly V366, G369, and Y370 (Figure 6), and also include alkyl-π interactions with I163, a key residue implicated in a conserved motif known to undergo conformational changes upon receptor activation.^25^

**Table 2:** Binding Free Energy (Δ*G*_tot_) of **(5)-(7)** to the 5-HT_2A_R Inside the Orthosteric Binding Pocket and Decomposition into Ligand/Receptor van der Waals (Δ*G*_vdw_) and Electrostatic (Δ*G*_el_) Interactions, and Complex/Solvent Polar (Δ*G*_polar_) and Non Polar (Δ*G*_np_) Interactions in kcal/mol

compounds	Δ*G*_vdw_	Δ*G*_el_	Δ*G*_pol_	Δ*G*_np_	Δ*G*_tot_
25CN-N1-Nap **(5)**	–63.6	–103.4	102.3	–7.3	–72.2
25CN-NBPh **(6)**	–62.4	–104.8	108.1	–7.4	–67.0
25CN-NDBF **(7)**	–67.9	–101.7	102.1	–7.9	–75.5

#### Binding Free Energy Associated with the Toggle Switch

To further investigate the driving forces behind the formation of the two distinct minima observed in the US simulations, we performed an MMGBSA analysis, treating the toggle switch W336 as the ligand and the rest of the protein along with compound **(7)** as the receptor. The geometries for the free energy calculations were extracted at equidistant intervals from the 50 ns simulations corresponding to the two US windows where the free-energy minima were identified. We specifically focused on the US simulation of compound **(7)**, as it presented the largest energy difference between the two minima. This approach allowed us to compute Δ*G*_tot_ between W336 and the rest of the system, and decompose it into pairwise interactions to identify the key components responsible for its stabilization. The major energy difference is observed in the Δ*G*_vdw_, which favors the global minimum at χ_2_(W336) = −5°, resulting in a more favorable Δ*G*_tot_. Both Δ*G*_el_ and Δ*G*_pol_ also favor the global minimum, while Δ*G*_np_ remains unchanged between the two conformations, as seen in Table 3.

**Table 3:** Binding Free Energy (Δ*G*_tot_) of W336 at χ_2_(W336) at −5° and 95° Inside the Orthosteric Binding Pocket and Decomposition into Ligand/Receptor van der Waals (Δ*G*_vdw_) and Electrostatic (Δ*G*_el_) Interactions, and Complex/Solvent Polar (Δ*G*_pol_) and Polar (Δ*G*_np_) Interactions in kcal/mol

25CN-NDBF **(7)**	Δ*G*_vdw_	Δ*G*_el_	Δ*G*_pol_	Δ*G*_np_	Δ*G*_tot_
χ_2_(W336) = −5°	–61.2	–29.6	21.1	–3.9	–69.0
χ_2_(W336) = 95°	–55.4	–27.1	22.0	–4.0	–60.0

Pairwise binding free energy decomposition identifies F332 as the major contributor to the stabilization of W336 in both conformations at χ_2_(W336) = −5° and χ_2_(W336) = 95°, with a slightly stronger Δ*G*_vdW_ for the latter (Figure 7A). Visual inspection of the 50 ns of MD simulation in both χ_2_(W336) = −5° and χ_2_(W336) = 95° US windows displays interesting behavior of F332 (Figure 7B). In the window corresponding to χ_2_(W336) = −5° the χ_2_(F332) is stabilized in a conformation at approximately −30°, while in the window corresponding to the US window at χ_2_(W336) = 95° a large change in displacement is observed for χ_2_(F332), shifting to 180°. In addition, in both minima, the toggle switch interacts with I163 primarily through Δ*G*_vdw_ interactions, but no clear conformational changes in this residue were observed during the dynamics. Both F332 and I163 have been implicated as part of a motif explaining how allosteric changes are transmitted down the receptor,^25,28^ and these results underscore the importance of these residues and quantify their interactions with the toggle switch.

**Figure 7:**
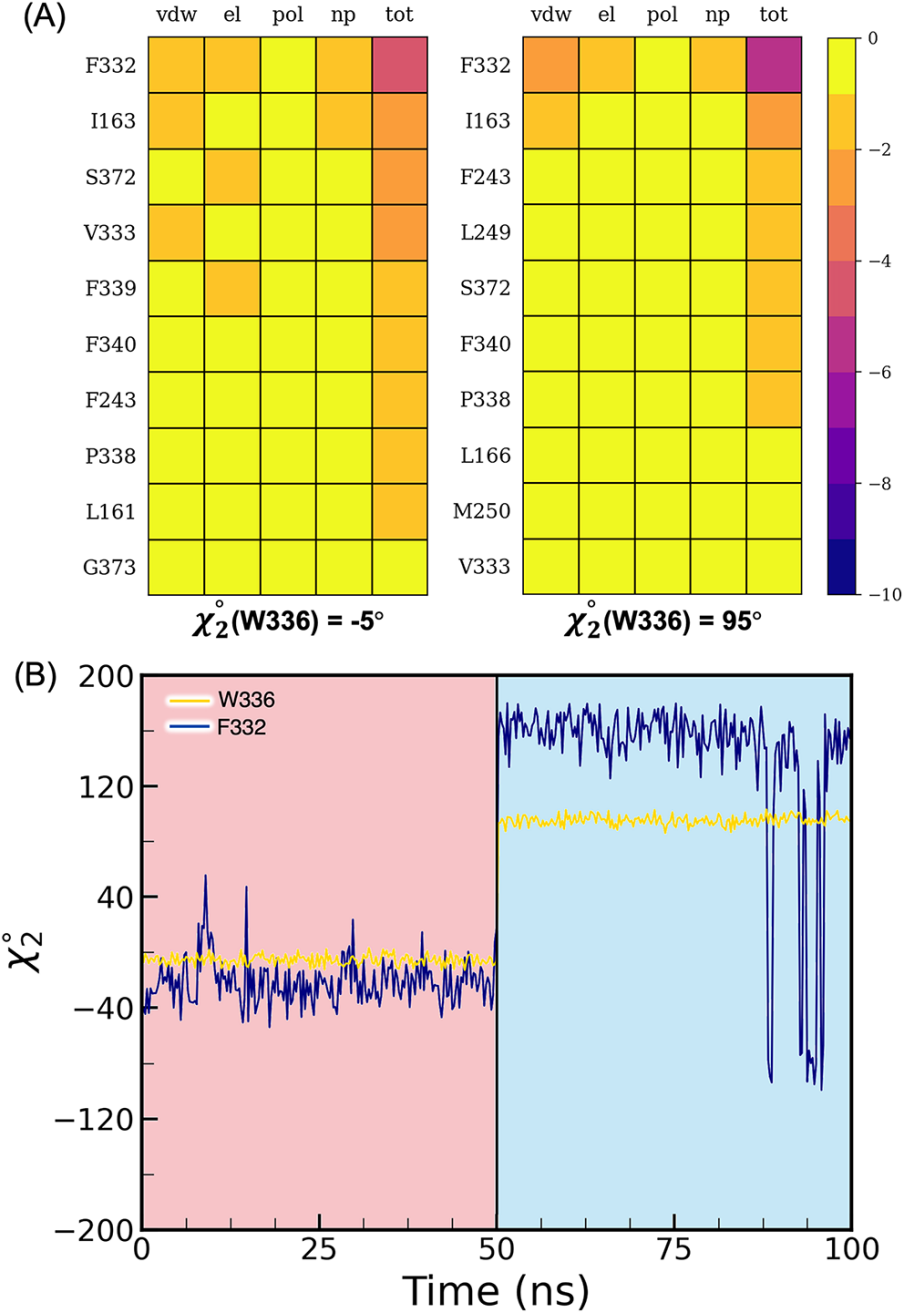
(A) Pairwise residue decomposition of the binding free energy for W336. Each residue contribution is in turn decomposed into ligand/protein van der Waals (vdw) and electrostatic (el) contributions and polar (pol) and nonpolar (np) solvation contributions to the total residue binding energy (tot). (B) Time evolution of the χ_2_ dihedral angle of F332 (yellow) and W336 (blue) during the umbrella sampling window at χ_2_(W336) = −5° (pink) and the umbrella sampling window at χ_2_(W336) = 95° (light blue).

## Conclusions

Determining which 5-HT_2A_R downstream signaling pathway is responsible for the hallucinatory effects of psychedelic compounds is essential for developing new treatments for neuropsychiatric disorders. Recent studies on a series of 25CN-NBx derivatives have revealed a direct correlation between hallucinatory effects in mice and the level of G_q/11_ pathway activation, with compounds exceeding 70% G_q/11_ efficacy consistently inducing psychedelic-like behaviors. This pathway activation has also been closely associated with conformational changes in the toggle switch W336. In this work, we demonstrate through a combination of unbiased and US MD simulations, together with MMGBSA calculations, that the stability and conformational changes of the toggle switch W336 can be predicted and tuned with different *N*-benzyl substitutions on 25CN-NBx compounds. Unbiased MD simulations indicate that W336 can naturally adopt two distinct conformations depending on the *N*-benzyl additions of the ligands, with the χ_2_(W336) serving as the key reaction coordinate relevant to this conformational change. Consequently, we employed US to sample χ_2_(W336) from −60° to 120° and found that the addition of bulky substituents to the *N*-benzyl group shifts the minimum energy of χ_2_(W336) to negative values, causing the indole core of W336 to orient toward the intracellular region of the 5-HT_2A_R. Indeed, the PMFs for **(5)**, **(6)**, and **(7)**, all of which exhibited low G_q/11_ efficacy and lacked hallucinogenic effects, showed a pronounced minimum at negative χ_2_(W336) values, confirming a correlation between reduced G_q/11_ signaling and the inward displacement of the toggle switch. The MMGBSA results align with experimental findings, showing that the addition of a −2-OH-3-Me group in compound **(3)** significantly enhances its affinity for the 5-HT_2A_R, whereas the −2-OH-5-MeO substitution in compound **(4)** results in the lowest affinity. The methyl group at the C_3′_ position appears to increase the electron density and promote a tighter fit within the hydrophobic regions of the binding pocket, leading to more favorable Δ*G*_vdw_ interactions with surrounding residues. In contrast, the methoxy group at C_5′_ does not enhance Δ*G*_el_ or Δ*G*_vdw_ but significantly increases the Δ*G*_pol_ penalty. The addition of bulky substituents, as in compounds **(5)**, **(6)**, and **(7)**, strengthens the van der Waals interactions overall, reflected by more favorable (i.e., more negative) Δ*G*_vdw_ values. Finally, we demonstrated that the stability of χ_2_(W336) at negative values is associated with stronger Δ*G*_vdw_ interactions, where the residue directly interacts with F332, stabilizing it at a distinct χ_2_(F332) conformation, and also forms strong contacts with I163. Both F332 and I163 have been implicated in the allosteric activation of the 5-HT_2A_R. These findings provide atomic-level insights into how different substituents within the 25CN-NBx class can stabilize specific conformations of the 5-HT_2A_R, and have broader implications for the study of GPCR activation mechanisms involving the TM6 toggle switch.

## Supplementary Material



## Notes

Computational details, molecular dynamics simulations, umbrella sampling methods, and MM/GBSA free energy analysis for 25CN-NBx compounds (PDF)
