# Supplementary material for: Rewiring Receptor Activation: Mechanistic Insights into Toggle Switch Modulation by 25CN-NBx Compounds
Source: ACS Chem Neurosci. 2026 Feb 18;17(5):1043–53. doi: 10.1021/acschemneuro.6c00023 (PMC12964406; doi:10.1021/acschemneuro.6c00023)
Supplement: Supplementary file 1 [file cn6c00023_si_001.pdf]

Supporting Information for Rewiring Receptor Activation: Mechanistic Insights into Toggle Switch Modulation by 25CN-NBx Compounds

Vito F. Palmisano,[*,†,‡] Micaela Vidal–Sánchez,[†] and Juan J. Nogueira[*,†,¶]

†*Department of Chemistry, Universidad Autonoma de Madrid, Madrid, Spain.*

‡*International Foundation Big Data and Artificial Intelligence for Human Development, Bologna, Italy*

¶*IADCHEM, Institute for Advanced Research in Chemistry, Universidad Autonoma de Madrid, Madrid, Spain*

E-mail: vito.palmisano@ifabfoundation.org; juan.nogueira@uam.es

Computational Details

Initial Structures

The holo-structure of the 5-HT_{2A}R bound to the selective agonist **(2)** was obtained from the RCSB-PDB (PDB ID: 6WHA).[1] Missing extracellular loops were filled using chain B of the holo-structure of the 5-HT_{2A}R bound to LSD (PDB ID: 6WGT), and missing heavy atoms and hydrogens were added using the tleap module of the AMBER22 package.[2] For **(2)** and all substituted compounds under investigation, the predominant titration state in an aqueous solution at neutral pH is the protonated form ($pK_a > 7.8$); thus, this state was selected for all simulations.[3] The geometries were optimized at the B3LYP/cc-pVDZ level of theory[4,5] and used to calculate the restricted electrostatic potential charges at the

Hartree-Fock/6-31G* level of theory, ensuring consistency within the AMBER force field.[6] All ligands were placed into the orthosteric binding pocket by aligning their shared backbone to the crystallized reference compound (2). Since all compounds have the same backbone, it is assumed that they will bind in a similar way into the protein. The N-benzyl substitutions were thus introduced in a consistent manner, and subsequent MD simulations allowed both the ligands and receptor to adapt, ensuring realistic binding poses without the need for docking. All membrane-protein-ligand systems were constructed using CHARMM-GUI.[7] The protein was oriented along the z-axis using the orientations of proteins in membranes database and the positioning of proteins in membrane web server.[8] Subsequently, N- and C-termini were amidated and acetylated. The resulting structure was then embedded within a 1-palmitoyl-2-oleoyl-*sn*-glycero-3-phosphocholine lipid bilayer with dimensions of 50 x 50 lipid components in the xy plane and solvated in a rectangular box with aqueous solvent and NaCl at a concentration of 0.15 mol/L. Potential parameters for the protein, lipids, water, and ligands were taken from FF19SB, Lipid21, TIP3P, and GAFF2, respectively.[9–12]

Classical Molecular Dynamics Simulations

Classical molecular dynamics (MD) simulations for the solvated membrane-protein-ligand systems were performed using the CUDA version of the AMBER20 package.[2,13] First, energy minimization was carried out with the steepest descent method for 5000 steps, followed by the conjugate gradient method for additional 5000 steps. Positional restraints were applied to the membrane-protein-ligand system, gradually decreasing from 10 to 0.5 kcal/(mol Å^2), while heating from 0 to 303.15 K using the Langevin thermostat for a total of 750 ps. The desired density was reached by running an equilibration in the NPT ensemble with a Monte Carlo barostat and semi-isotropic pressure scaling for 500 ps. An unconstrained production run was carried out at 303.15 K for 100 ns, followed by 4 independent copies of 1 μs (4 μs per ligand) for a total simulation time of 28 μs. During the full MD protocol, a timestep of 2 fs was employed. The cutoff radius and switching distance for computing the non-

bonded interactions were set to 10.0 Å and 9.0 Å, respectively, and bond lengths involving hydrogen atoms were kept fixed using the SHAKE algorithm.[14] Electrostatic interactions were calculated using the particle-mesh Ewald method with a grid spacing of 1 Å.[15]

Umbrella Sampling Simulations

In some replicas of the unbiased MD simulations, W336 was observed to naturally transition from its initial crystallized state with a χ_2(W336) dihedral angle of $\approx$ 70° to $\approx$ -20°. Using the same setup previously described, a representative geometry from the MD simulation was used to start a pulling run, scanning the χ_2(W336) dihedral angle (reaction coordinate) from 120° to -40° employing a pulling rate of 1°/ns for a total of 160 ns for each compound **(1)-(7)**. The acquired initial geometries were employed to run umbrella sampling (US) simulations with a total of 33 windows evenly spaced 5 degrees apart, to ensure adequate overlap between neighboring distributions. A harmonic restraint with a force constant of 90.0 kcal/mol/rad^2 was applied to maintain the value of the reaction coordinate, and for some high energy window, a stronger bias potential with a force constant of 150.0 kcal/mol/rad^2 was employed. For each compound and in each window, a MD trajectory was run for 50 ns, resulting in a total simulation time of 11.55 μs. The weighted histogram analysis method (WHAM) was then employed to calculate the potential of mean force (PMF) along the reaction coordinate using data obtained from each US simulation.[16,17]

MMGBSA Free Energy Analysis

The relative binding free energy, ΔG_{tot}, decomposed into electrostatic (ΔG_{el}), van der Waals (ΔG_{vdw}), polar solvation (ΔG_{pol}), and non-polar solvation (ΔG_{np}), was calculated for all the ligand-receptor complexes using the 1A-molecular mechanics generalize Born surface area end-state method.[18] A total of 200 equidistant geometries were obtained from the US window corresponding to the global minimum energy for each complex, and a pairwise residue decomposition analysis was performed to further decompose each of the terms into

residue-ligand free energies. This allowed for the quantification and characterization of the interactions upon N-benzyl substitution. The same procedure was carried out by treating W336 as the ligand within the ligand-receptor complex, considering the rest of the protein and the 25CN-NBx molecules as the receptor, to characterize the interactions of W336 at the two minima for the bulky compounds **(5)-(7)**.

References

(1) Kim, K.; Che, T.; Panova, O.; DiBerto, J. F.; Lyu, J.; Krumm, B. E.; Wacker, D.; Robertson, M. J.; Seven, A. B.; Nichols, D. E. Structure of a hallucinogen-activated Gq-coupled 5-HT2A serotonin receptor. *Cell* **2020**, *182*, 1574–1588. e19.

(2) Case, D. A.; Aktulga, H. M.; Belfon, K.; Ben-Shalom, I.; Brozell, S. R.; Cerutti, D. S.; Cheatham III, T. E.; Cruzeiro, V. W. D.; Darden, T. A.; Duke, R. E. *Amber 2021*; University of California, San Francisco, 2021.

(3) Wishart, D. S.; Feunang, Y. D.; Guo, A. C.; Lo, E. J.; Marcu, A.; Grant, J. R.; Sajed, T.; Johnson, D.; Li, C.; Sayeeda, Z.; others DrugBank 5.0: a major update to the DrugBank database for 2018. *Nucleic Acids Res.* **2018**, *46*, D1074–D1082.

(4) Becke, A. D. Density-functional thermochemistry. III. The role of exact exchange. *J. Chem. Phys.* **1993**, *98*, 5648–5652.

(5) Dunning Jr, T. H. Gaussian basis sets for use in correlated molecular calculations. I. The atoms boron through neon and hydrogen. *J. Chem. Phys.* **1989**, *90*, 1007–1023.

(6) Bayly, C. I.; Cieplak, P.; Cornell, W.; Kollman, P. A. A well-behaved electrostatic potential based method using charge restraints for deriving atomic charges: the RESP model. *J. Phys. Chem.* **1993**, *97*, 10269–10280.

(7) Jo, S.; Kim, T.; Iyer, V. G.; Im, W. CHARMM-GUI: a web-based graphical user interface for CHARMM. *J. Comput. Chem.* **2008**, *29*, 1859–1865.

(8) Lomize, M. A.; Pogozheva, I. D.; Joo, H.; Mosberg, H. I.; Lomize, A. L. OPM database and PPM web server: resources for positioning of proteins in membranes. *Nucleic Acids Res.* **2012**, *40*, D370–D376.

(9) Tian, C.; Kasavajhala, K.; Belfon, K. A.; Raguette, L.; Huang, H.; Migues, A. N.; Bickel, J.; Wang, Y.; Pincay, J.; Wu, Q. ff19SB: Amino-acid-specific protein backbone parameters trained against quantum mechanics energy surfaces in solution. *J. Chem Theory Comput.* **2019**, *16*, 528–552.

(10) Gould, I.; Skjevik, A.; Dickson, C.; Madej, B.; Walker, R. Lipid17: A comprehensive AMBER force field for the simulation of zwitterionic and anionic lipids. *Manuscript in preparation* **2018**,

(11) Jorgensen, W. L.; Chandrasekhar, J.; Madura, J. D.; Impey, R. W.; Klein, M. L. Comparison of simple potential functions for simulating liquid water. *The Journal of Chemical Physics* **1983**, *79*, 926–935.

(12) Wang, J.; Wolf, R. M.; Caldwell, J. W.; Kollman, P. A.; Case, D. A. Development and testing of a general amber force field. *J. Comput. Chem.* **2004**, *25*, 1157–1174.

(13) Salomon-Ferrer, R.; Gotz, A. W.; Poole, D.; Le Grand, S.; Walker, R. C. Routine microsecond molecular dynamics simulations with AMBER on GPUs. 2. Explicit solvent particle mesh Ewald. *J. Chem. Theory. Comput.* **2013**, *9*, 3878–3888.

(14) Ryckaert, J.-P.; Ciccotti, G.; Berendsen, H. J. Numerical integration of the cartesian equations of motion of a system with constraints: molecular dynamics of n-alkanes. *J. Comput. Phys.* **1977**, *23*, 327–341.

(15) Darden, T.; York, D.; Pedersen, L. Particle mesh Ewald: An N log (N) method for Ewald sums in large systems. *J. Chem. Phys.* **1993**, *98*, 10089–10092.

(16) Kumar, S.; Rosenberg, J. M.; Bouzida, D.; Swendsen, R. H.; Kollman, P. A. The weighted histogram analysis method for free-energy calculations on biomolecules. I. The method. *J. Comput. Chem.* **1992**, *13*, 1011–1021.

(17) Grossfield, A.; Woolf, T. B. Interaction of tryptophan analogs with POPC lipid bilayers investigated by molecular dynamics calculations. *Langmuir* **2002**, *18*, 198–210.

(18) Miller III, B. R.; McGee Jr, T. D.; Swails, J. M.; Homeyer, N.; Gohlke, H.; Roitberg, A. E. MMPBSA. py: an efficient program for end-state free energy calculations. *J. Chem. Theory Comput.* **2012**, *8*, 3314–3321.
